# EBV+ and Kaposi’s Sarcoma Herpesvirus‐Associated Multicentric Castleman Disease in a Patient With HIV Infection: A Case Report

**DOI:** 10.1155/crdi/5567369

**Published:** 2026-01-02

**Authors:** Mai-Yin Huang, Zhe Li, Wei Zhang, Hai-Yun Chen, Jun Liu, Chong-Xi Li

**Affiliations:** ^1^ Department of Dermatology, First Affiliated Hospital of Kunming Medical University, Kunming, Yunnan, China, kmmc.cn; ^2^ Department of Infectious Disease, The Third People’s Hospital of Kunming (Yunnan Clinical Medical Center for Infectious Diseases), Kunming, Yunnan, China

**Keywords:** case report, Epstein-Barr virus, human immunodeficiency virus, Kaposi’s sarcoma herpesvirus, Multicentric Castleman disease, rituximab

## Abstract

Multicentric Castleman disease is a rare proliferative disease of lymphoid tissue. It has rarely been reported in Asian countries, particularly in HIV‐positive patients. Here, we report a case of Kaposi’s sarcoma herpesvirus‐associated Multicentric Castleman disease (KSHV‐MCD). A 44‐year‐old male HIV patient with a good response to antiretroviral therapy presented with recurrent fever and bilateral axillary masses. He was hospitalized for recurrent exacerbations, and it took 7 years from onset to definitive diagnosis. Lymph node biopsy suggested Castleman disease. Metagenomic next‐generation sequencing (mNGS) of the blood showed that the patient was infected with KSHV (8327 sequence reads) and EBV (283 sequence reads). The patient was administered rituximab, thalidomide, sodium phosphonates, and ganciclovir. The patient’s symptoms were completely relieved, and all indicators returned to normal, with no recurrence during follow‐up. This case underlines that it is necessary to perform multiple lymph node biopsies or repeat the biopsies multiple times for the diagnosis of KSHV‐MCD.

## 1. Introduction

Castleman disease (CD) is a rare lymphoproliferative disorder that was first reported by Castleman et al. in 1956 [1]. It is classified into unicentric Castleman disease (UCD) and Multicentric Castleman disease (MCD) based on clinical presentation and course. MCD presents with systemic, progressive, and life‐threatening symptoms, including recurrent systemic symptoms (weight loss, fever, and fatigue), hepatosplenomegaly, anemia, fatigue, multiple regional lymphadenopathies, and poorer prognosis than UCD [2, 3]. Although the precise etiology and pathogenesis remain elusive, previous studies have implicated Kaposi’s sarcoma herpesvirus (KSHV) infection as pivotal in its pathogenesis. KSHV encodes its viral cytokines, such as viral IL‐6 (vIL‐6), which is similar to human IL‐6, and they are implicated in lymph node hyperplasia and inflammation [4–6]. In 2014, Fajgenbaum et al. proposed further classifying MCD based on KSHV status into KSHV‐associated MCD (KSHV‐MCD) and KSHV‐negative MCD (iMCD) [7]. In a recent study, OS at 5 and 10 years for patients with KSHV‐MCD (without Kaposi sarcoma [KS]) was 90% and 73%. For those with both KSHV‐MCD and KS, OS at 10 years was 81% [4]. KSHV‐MCD tends to occur in HIV‐infected patients or immunosuppressed individuals. Little epidemiological data are available on CD, particularly in low‐ and middle‐income countries [8]. In Western countries, MCD cases are thought to be divided almost evenly between KSHV‐MCD and iMCD [9]. Conversely, reports from Asian countries, such as China and Japan, show an iMCD predominance, with relatively few reports on KSHV‐MCD [10, 11].

Here, we present a case of an HIV‐positive KSHV‐MCD patient from China who exhibited recurrent systemic symptoms, including fever, hepatosplenomegaly, decreased blood cells, and elevated IL‐6. The patient endured 7 years from onset to diagnosis and achieved clinical remission following treatment with rituximab, thalidomide, ganciclovir, and sodium phosphonate.

## 2. Case Presentation

**Figure 1:**
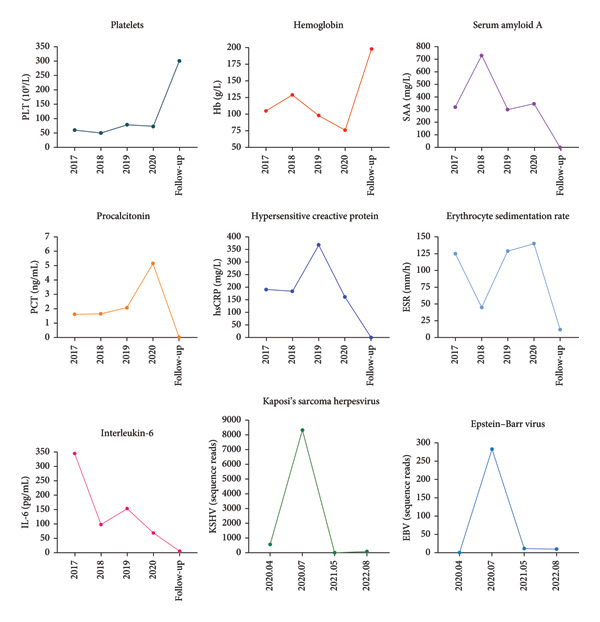
Some laboratory test results of the patient during hospitalization and follow‐up. The patient started treatment in July 2020. Platelets: PLT; Hb: hemoglobin; SSA: serum amyloid A; PCT: procalcitonin; hsCRP: hypersensitive C‐reactive protein; ESR: erythrocyte sedimentation rate; IL‐6: interleukin‐6.

**Figure 4:**
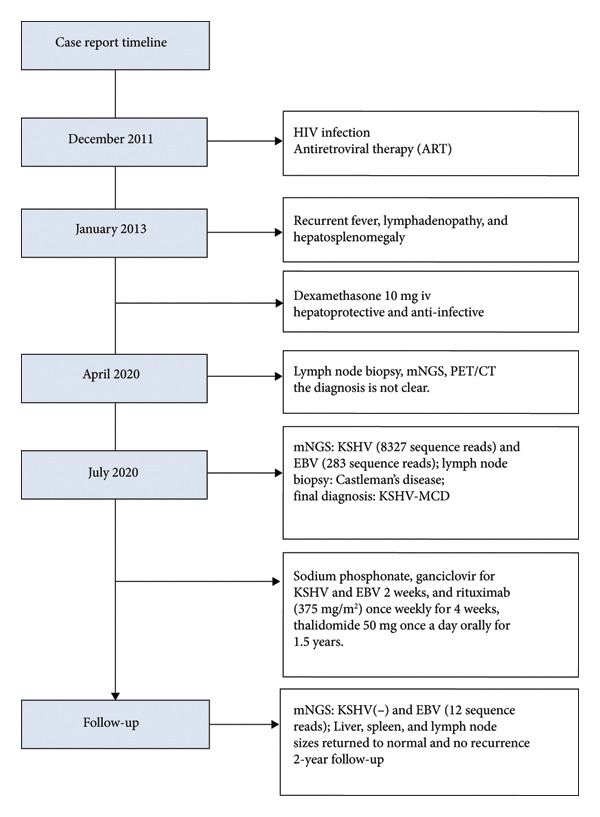
Diagnostic and treatment process of the case.

A 44‐year‐old male patient with HIV infection was referred to our department due to recurrent fevers and bilateral axillary masses. The patient was diagnosed with HIV infection in December 2011 and initiated antiretroviral therapy (ART) with the following regimen: zidovudine 300 mg every 12 h, lamivudine 300 mg once daily, and nevirapine 200 mg every 12 h. On February 21, 2012, because of hepatotoxicity, the regimen was modified to zidovudine 300 mg every 12 h, lamivudine 300 mg once daily, and lopinavir/ritonavir 500 mg every 12 h, and the viral load suppression was below the lowest detectable limit. Since 2013, he presented recurrent fever, with body temperatures of 38.5°C–39°C, and accompanied by generalized aches and fatigue, coughing, abdominal pain, diarrhea, axilla lymphadenopathy, and hepatosplenomegaly. Improvement can be achieved with antimicrobial and symptomatic treatment in private clinics. The fever recurred every 2–3 months and lasted for approximately 4–10 days each time. In 2015, a bone marrow biopsy was performed at other medical facilities with suspicion of lymphoma, and this diagnosis was excluded. Since 2017, he has been admitted to the Department of Hepatology multiple times. The abnormal test results during several hospitalizations are shown in Figure 1. Additionally, Epstein–Barr Virus (EBV) antibodies tested positive. Other tests showed no abnormalities, including cytomegalovirus (CMV)‐DNA and CMV antibodies, hepatitis B virus antibodies, hepatitis C virus antibodies, autoimmune hepatitis antibodies, brucella antibodies, and bone marrow smears and cultures, as well as blood cultures. Chest CT revealed nodular shadows in the middle lobe of the right lung and multiple nodular high‐density shadows in the lower lobes of both lungs. Abdominal CT showed multiple enlarged lymph node shadows in the abdominal cavity, bilateral axillae, and mediastinum, raising suspicion of lymphoma. Despite undergoing numerous examinations, a definitive diagnosis has yet to be established. Key observations included reductions in hemoglobin and platelet counts during fever, accompanied by elevated levels of C‐reactive protein (CRP), interleukin‐6 (IL‐6), and serum amyloid A (SSA). When the persistent high fever did not respond to treatment, dexamethasone 10 mg was administered via intravenous drip once daily for 3–4 days, which resulted in symptom relief. The patient was hospitalized almost every month. Each time the patient experienced disease flare‐ups, the CD4+ T‐cell count was markedly reduced. However, following remission, the count increased during ART follow‐up (Figure 2). In April 2020, the patient was transferred to our department for further treatment. Metagenomic next‐generation sequencing (mNGS) in blood showed that he was infected with KSHV (555 sequence reads). Positron emission tomography/computer tomography (PET/CT) suggested that lymphoma was very likely to be suspected. A biopsy of the right axillary lymph node was conducted, which showed follicular hyperplasia accompanied by conspicuous vascular proliferation in the interfollicular zones, with extensive plasma cell infiltration (Figure 3). Immunohistochemistry confirmed predominantly follicular zone cells CD20(+) and CD79a(+); germinal centers CD10(+), Bcl‐6(+), Bcl‐2(−), and Ki‐67(+, about 80%); follicular dendritic cells CD21(+) and CD23(+); interfollicular zone cells mostly CD2(+), CD3(+), CD5(+), and Ki‐67(+, about 20%); and plasma cells MUM1(+). Unfortunately, due to the rarity of the disease and the inexperience of clinicians and pathologists, a specific diagnosis remains elusive. He was given symptomatic treatment with blood transfusion, human albumin infusion, glucocorticoids, and anti‐infection. Due to recurrent anemia, the antiretroviral regimen was adjusted to include raltegravir. During this period, his viral load remained below the lower limit of detection. In July 2020, he was transferred to another hospital for hospitalization due to a fever, and during hospitalization, mNGS in blood was reviewed again, which showed that he was infected with KSHV (8327 sequence reads) and EBV (283 sequence reads). Pathological diagnosis showed that the (left cervical lymph node) Castleman’s disease was considered. Immunophenotypic analyses showed CD3 (T cell, +), CD20 (B cell,+), CD79*α* (B cell, +), CD21 (FDC network, +), CD43 (T cell, +), CD10 (follicle center, +), BCL‐2 (follicle center, −) BCL‐6 (follicle center, +), Ki67 (follicle center about 90% +, perifollicle about 30% +), CD30 (few, +), Pax‐5 (B cells, +), ALK1 (−), MUM1 (few +), CyclinD1 (few +), CD5 (T cells, +), CD19 (+), CD22 (+), KSHV (+), actin (vasculature,+), and PD‐1 (+), IgG4 (few, +). Molecular pathology results: EBER (+). The final diagnosis for the patient was KSHV‐MCD. The treatment involved sodium phosphonate (60 mg/kg/d, intravenous drip, once every 8 h),ganciclovir (5 mg/kg/d for KSHV and EBV, lasting 14 days), rituximab (375 mg/m2 once weekly for 4 weeks), combined with thalidomide (50 mg, once daily) for 1.5 years. The patient responded well to treatment and showed no recurrence at the 3‐year follow‐up. The diagnostic and treatment flowchart for the case is presented in Figure 4. Detailed post‐treatment follow‐up reports are provided in Figures 1 and 2 and in Table 1.

Figure 2(a) Each time the patient experienced disease flare‐ups, the CD4+ T‐cell count was markedly reduced. (b) Following remission, the count increased during ART follow‐up.

**Figure:**
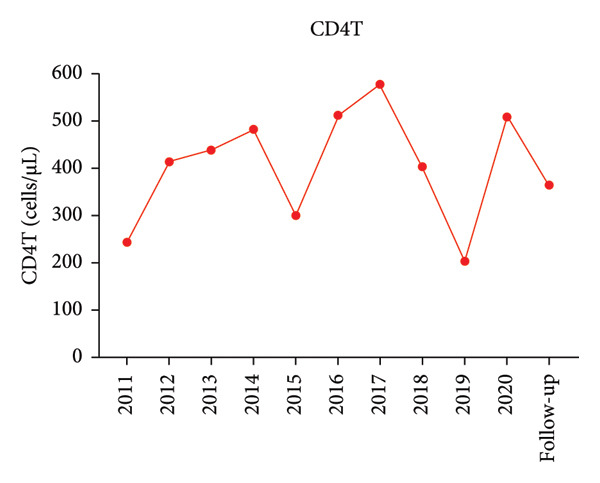
(a)

**Figure:**
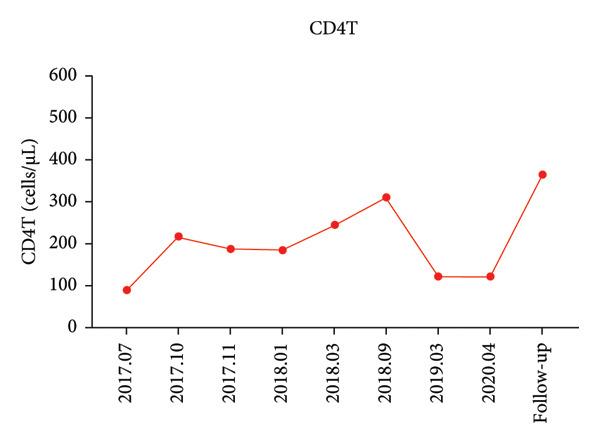
(b)

Figure 3(a) Hematoxylin and eosin stain, original magnification ×40. The lymph node architecture is preserved, showing prominent follicular hyperplasia with clearly defined follicular structures and interfollicular regions. (b, c) Hematoxylin and eosin stain, original magnification ×100. Follicular hyperplasia accompanied by conspicuous vascular proliferation in the interfollicular zones, with extensive plasma cell infiltration.

**Figure:**
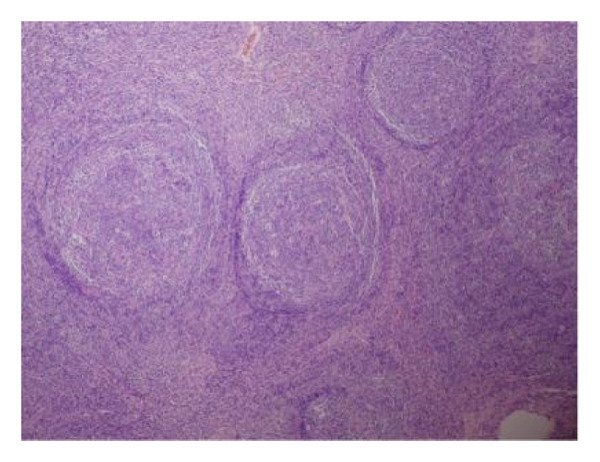
(a)

**Figure:**
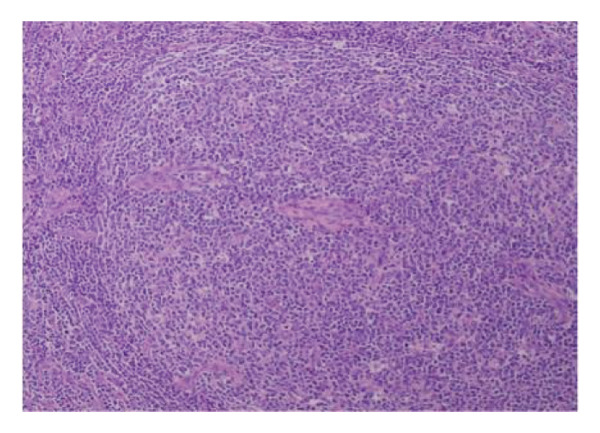
(b)

**Figure:**
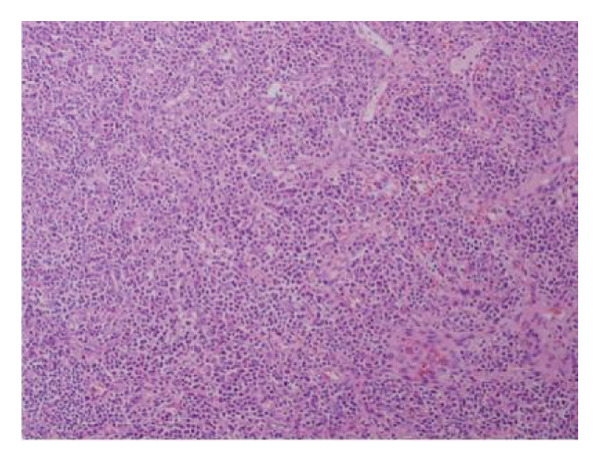
(c)

**Table 1:** Pretreatment and post‐treatment follow‐up report.

Item	Abdominal ultrasound	Lymph node ultrasound
July 2020	Marked splenomegaly (length: 176 mm; thickness: 62 mm)	Cervical: right largest 19.7 × 7 mm, left largest 17.8 × 8.2 mm Supraclavicular: right largest 13.3 × 4.9 mm, left largest 15 × 5.3 mm

May 2021	Spleen size decreased (length: 143 mm; thickness: 46 mm)	Cervical: right largest 23 × 8 mm, left largest 21 × 8 mm. Axillary: right largest 22 × 5 mm, left largest 19 × 6 mm

August 2021	Spleen size further reduced (length: 119 mm; thickness: 40 mm)	Cervical: right largest 10 × 6 mm, left largest 10 × 5 mm

## 3. Discussion

KSHV‐MCD is currently considered to be a rare lymphoproliferative disorder. In Western countries, KSHV‐MCD frequently occurs in HIV‐infected individuals [12]. However, KSHV‐MCD is rarely observed in HIV‐positive patients in China or other Asian countries, and only a few cases have been reported to date. A nationwide, multicenter, retrospective study from China included 731 patients with MCD, among whom only 12 patients were infected with KSHV, and all patients tested negative for HIV [10], consistent with data previously reported in Japan [11]. The reasons for this observation remain elusive and may be influenced by regional HIV variations. This underscores the rarity of this case. Although KSHV‐MCD is considered rare, it may be observed more commonly in the era of ART and frequently occurs in HIV patients with well‐maintained immune function [13]. Our case presented with a low CD4 count of only 64 cells/μL at disease onset. The pathogenesis of KSHV‐MCD is thought to be associated with the dynamics of KSHV, which is characterized by a two‐phase lifecycle. During the latent phase, the virus remains dormant and expresses only a limited number of genes. However, during the lysis cycle, HIV infection‐associated immunosuppression enables the immune escape of KSHV, which aids viral lysis and replication in the lymph nodes. The replicating virus expresses various lytic proteins and triggers upregulation of human IL‐6 (huIL‐6), vIL‐6, and other cytokines [14, 15]. Both vIL‐6 and hIL‐6 promote the proliferation of B cells, thereby activating systemic inflammation and lymph node hyperplasia. During an inflammatory flare, the KSHV load in the blood increases [16]. The KSHV viral load and serum cytokines correlate with recurrent systemic symptoms, effusion, and laboratory abnormalities, including decreased blood cells, hypoalbuminemia, and elevated inflammatory markers [17]. We posit a potential correlation between cyclical recurrence and relief noted in patients with KSHV‐MCD and the latent and lytic phases of KSHV.

Notably, the patient was infected not only with KSHV but also with EBV. A cooperative viral mechanism exists between EBV, KSHV, and HIV‐1, which contributes to the development of various tumors. KSHV augments EBV‐associated tumorigenesis by stimulating lytic EBV replication [18]. Cases of MCD concurrently infected with EBV and KSHV are infrequent, with only a few case reports. For instance, one patient developed hemophagocytic syndrome after surgical intervention, which ultimately resulted in a fatal outcome [19]. In addition, another patient responded well to treatment with rituximab [20]. We suggest that our case might result incidentally from the higher EBV infection rate in the local population, or the infection could be triggered by the immunological abnormalities of MCD. However, further research is necessary to determine its role as a causative factor for MCD.

Confirmation of CD diagnosis requires a pathological review of the affected lymph node, which should ideally be performed from an excisional biopsy rather than relying solely on a needle biopsy. However, the rarity of this disease presents a diagnostic challenge, particularly for inexperienced pathologists. It is widely recognized that in patients with recurrent fever, pathogenic microorganism inspection commonly does not include KSHV detection. Nevertheless, a case report showed that metagenomic sequencing (MGS) technology can provide clues for disease diagnosis [21]. We performed mNGS and lymph node biopsy of the patient, which revealed KSHV infection but did not achieve a definitive diagnosis of MCD. Recent studies have indicated that even in the absence of pathognomonic features of KSHV‐MCD, consideration of KSHV‐associated diseases is warranted when HIV patients are coinfected with KSHV associated with the patient’s clinical symptoms [13]. KSHV is implicated in several important clinical conditions, including KS, primary effusion lymphoma, MCD, KSHV inflammatory cytokine syndrome (KICS), and KSHV immune reconstitution syndrome (KS‐IRIS) [22]. KICS and MCD share similar clinical presentations and laboratory findings, and their differentiation relies primarily on pathological examination. KICS lacks the distinctive histopathological feature characteristic of MCD. KICS was first defined by Polizzotto et al. [23] and includes (1) clinical manifestations: (a) symptoms (fever, fatigue, and edema), (b) laboratory abnormalities (anemia, thrombocytopenia, hypoalbuminemia, and hyponatremia), and (c) imaging findings (lymphadenopathy, hepatosplenomegaly, and effusions); (2) evidence of systemic inflammation: elevated CRP (≥ 3 g/dL); (3) evidence of KSHV viral activity; and (4) the absence of evidence of MCD on histopathologic review of tissue from lymph node biopsy. Diagnosis requires the presence of at least 2 clinical manifestations drawn from at least 2 categories (1a, b, and c), together with each of the criteria in 2, 3, and 4. The patient presented with clinical manifestations consistent with the diagnostic criteria for KICS, including constitutional symptoms (fever, fatigue, and respiratory symptoms), laboratory abnormalities (anemia, thrombocytopenia, and hypoalbuminemia), and elevated CRP and evidence of KSHV viral activity. However, the initial lymph node biopsy and immunohistochemical findings were in fact consistent with the pathological features of MCD, though the diagnosis was not established at that time due to limited clinical and pathological experience. Despite the lack of pathological evidence of KSHV‐MCD in our patient, the combination of clinical presentation and laboratory tests should have raised a high suspicion of KICS. Presently, the treatment modalities for MCD and KICS are similar. Nevertheless, KICS remains insufficiently recognized among clinicians and pathologists, and its true incidence is likely substantially higher than currently reported. Clinicians may have encountered KICS before, but most are unaware of the entity and how to make the diagnosis [24, 25]. KICS is usually seen in patients who have poorly controlled HIV and low CD4 counts [26]. Research indicates that in HIV and KSHV coinfected patients, HIV is considered to directly promote KSHV replication, while decreased CD4 counts and cytokine secretion indirectly contribute to the KSHV replication [27, 28]. KSHV‐MCD is most common in patients with suppressed HIV and preserved CD4 counts [29]. However, several studies have indicated that the development of KSHV‐MCD is not associated with CD4 T‐cell levels [13]. Our case presented with a low CD4 count of only 64 cells/μL at disease onset and exhibited a further decline during each symptomatic episode, while HIV RNA levels remained consistently below 50 copies/mL. Ultimately, regardless of a patient’s CD4 T‐cell level, histopathologic examination of lymph node biopsy remains the definitive method to distinguish KICS from KSHV‐MCD. Our definitive diagnosis was established only after a secondary lymph node biopsy, highlighting the necessity for repetitive biopsies or biopsies taken from multiple sites. This poses a challenge for clinicians because of its rarity, often resulting in diagnostic delays, diagnostic confusion, and treatment delays, which significantly affect the patients’ quality of life. Therefore, enhancing awareness of this disease among clinicians and pathologists is paramount.

The patient experienced multiple challenges during the diagnostic process, and numerous visits failed to identify the exact cause. It took 7 years from onset to diagnosis. This case provides a valuable lesson: even if KSHV infection is not conclusively diagnosed pathologically, ganciclovir should be considered for antiviral treatment. Reports have indicated that antiviral therapy can effectively inhibit KSHV replication and alleviate symptoms [30]. Additionally, timely measures for Multi‐Disciplinary Treatment (MDT) are essential. Untreated KSHV‐MCD may lead to phagocytic syndrome or fatality, multiple organ failure, infection, or lymphoma progression [31]. The use of rituximab has significantly improved prognosis, as evidenced by an increase in the 5‐year overall survival rate from 33% to 90% [29], facilitating the conversion of lethal diseases into chronic relapsing and remitting conditions. Thalidomide exhibits anti‐inflammatory, immunomodulatory, and antiangiogenic effects, contributing to a reduction in IL‐6 levels. When combined with rituximab, it is considered safe and well‐tolerated in clinical practice [32]. Our patient received treatment with rituximab, thalidomide, sodium phosphonate, and ganciclovir, resulting in remission. Recurrence was not observed at the 2‐year follow‐up visit, and the KSHV serological test results were negative. Even so, studies have shown that relapse is not uncommon after rituximab treatment and may occur after the restoration of CD19 B‐cell counts. Despite the use of rituximab, the incidence of HIV‐associated lymphoma is three times higher than that in the general population of patients with HIV. Most patients achieved remission with rituximab at the time of relapse. Clinical follow‐up should be conducted every 3–6 months to increase patient awareness regarding the risk of relapse [33].

## 4. Conclusion

The possibility of KSHV‐associated diseases should be considered in patients with HIV infection who present with periodic fever, fatigue, anemia, hepatosplenomegaly, and lymphadenopathy. In the ART era, the incidence of KSHV‐MCD appears to be increasing; therefore, clinicians and pathologists should enhance their awareness of this disease and implement timely MDT measures. This case highlights the necessity of repeat or multiple lymph node biopsies during the diagnostic process. Additionally, mNGS may provide valuable information for disease diagnosis. In the event of disease relapse, rituximab remission can be repeatedly used, and it is critical to provide health education to increase awareness of disease relapse and the risk of HIV‐associated lymphoma. Moreover, MCD treatments are frequently intense and prolonged, and some patients require lifelong maintenance therapy.

## Consent

Informed written consent was obtained from the patient for publication of this report and any accompanying images.

## Disclosure

The authors have read the International Committee of Medical Journal Editors (ICMJE) recommendations, and the manuscript was prepared and revised according to the ICMJE. All authors have read and approved the final manuscript.

## Conflicts of Interest

The authors declare no conflicts of interest.

## Author Contributions

Mai‐Yin Huang and Zhe Li contributed to manuscript writing and editing; Wei Zhang and Hai‐Yun Chen contributed to data collection; Jun Liu and Chong‐Xi Li contributed to conceptualization and supervision; Mai‐Yin Huang and Zhe Li authors contributed equally.

## Funding

This work was supported by the Kunming Health Commission Health Research Project (2020‐03‐08‐114) and the​ Project of the AIDS Bureau of Yunnan Province.

## Data Availability

The data that support the findings of this study are available from the corresponding author upon reasonable request.
